# *Plasmodium* parasites mount an arrest response to dihydroartemisinin, as revealed by whole transcriptome shotgun sequencing (RNA-seq) and microarray study

**DOI:** 10.1186/s12864-015-2040-0

**Published:** 2015-10-21

**Authors:** Philip J. Shaw, Sastra Chaotheing, Pavita Kaewprommal, Jittima Piriyapongsa, Chayaphat Wongsombat, Nattida Suwannakitti, Pongpisid Koonyosying, Chairat Uthaipibull, Yongyuth Yuthavong, Sumalee Kamchonwongpaisan

**Affiliations:** Protein-Ligand Engineering and Molecular Biology Laboratory, Medical Molecular Biology Research Unit, National Center for Genetic Engineering and Biotechnology (BIOTEC), National Science and Technology Development Agency (NSTDA), 113 Thailand Science Park, Thanon Phahonyothin, Tambon Khlong Neung, Amphoe, Khlong Luang, Pathum Thani 12120 Thailand; Biostatistics and Bioinformatics Laboratory, Genome Technology Research Unit, National Center for Genetic Engineering and Biotechnology (BIOTEC), National Science and Technology Development Agency (NSTDA), 113 Thailand Science Park, Thanon Phahonyothin, Tambon Khlong Neung, Amphoe, Khlong Luang, Pathum Thani 12120 Thailand

**Keywords:** Dihydroartemisinin, RNA-seq, Microarray, *Plasmodium*, Malaria, Transcriptome

## Abstract

**Background:**

Control of malaria is threatened by emerging parasite resistance to artemisinin and derivative drug (ART) therapies. The molecular detail of how *Plasmodium* malaria parasites respond to ART and how this could contribute to resistance are not well understood. To address this question, we performed a transcriptomic study of dihydroartemisinin (DHA) response in *P. falciparum* K1 strain and in *P. berghei* ANKA strain using microarray and RNA-seq technology.

**Results:**

Microarray data from DHA-treated *P. falciparum* trophozoite stage parasites revealed a response pattern that is overall less trophozoite-like and more like the other stages of asexual development. A meta-analysis of these data with previously published data from other ART treatments revealed a set of common differentially expressed genes. Notably, ribosomal protein genes are down-regulated in response to ART. A similar pattern of trophozoite transcriptomic change was observed from RNA-seq data. RNA-seq data from DHA-treated *P. falciparum* rings reveal a more muted response, although there is considerable overlap of differentially expressed genes with DHA-treated trophozoites. No genes are differentially expressed in DHA-treated *P. falciparum* schizonts. The transcriptional response of *P. berghei* to DHA treatment *in vivo* in infected mice is similar to the *P. falciparum in vitro* culture ring and trophozoite responses, in which ribosomal protein genes are notably down-regulated.

**Conclusions:**

Ring and trophozoite stage *Plasmodium* respond to ART by arresting metabolic processes such as protein synthesis and glycolysis. This response can be protective in rings, as shown by the phenomenon of dormancy. In contrast, this response is not as protective in trophozoites owing to their commitment to a highly active and vulnerable metabolic state. The lower metabolic demands of schizonts could explain why they are less sensitive and unresponsive to ART. The ART response pattern is revealed clearly from RNA-seq data, suggesting that this technology is of great utility for studying drug response in *Plasmodium*.

**Electronic supplementary material:**

The online version of this article (doi:10.1186/s12864-015-2040-0) contains supplementary material, which is available to authorized users.

## Background

Control, and ultimately eradication of malaria require effective drugs to treat infections. The current most widely-used artemisinin-based combination therapies are being undermined by the emergence of resistance in Southeast Asia. Isolates from different *P. falciparum* parasite populations displaying slow clearance in patients possess 10–20 fold greater tolerance to artemisinin and derivatives with the same pharmacophore (ART) *in vitro* [1, 2].

Following the completion of the *P. falciparum* genome sequence and the development of microarray technology for interrogating the transcriptome, several studies were published of parasite transcriptomic response to perturbation, including anti-malarial drugs. Whereas parasite response to some drugs such as antifolates is modest [3], other drugs, including ART provoke a greater response [4, 5]. Greater understanding of the parasite transcriptional response to ART could give insight into the drug’s mode of action and the mechanism of parasite resistance, since ART-resistant parasites display altered transcriptional programs during the normal intra-erythrocytic development cycle (IDC), as shown by microarray analysis of slow-clearance field isolates [6, 7] and laboratory-induced resistant parasites [8, 9]. Interpretation of the ART transcriptional response is complicated by the fact that sensitivity to this class of drug varies greatly through the IDC; trophozoites are the most sensitive, whereas other stages are up to 100-fold less sensitive [10–12]. This variation could correspond with different transcriptional responses depending on the stage of parasite developmental.

A previous microarray study of artesunate response in *P. falciparum* trophozoites revealed a major transcriptional response to the drug [4]. However, the question of whether other stages of parasite development would respond in a similar fashion was not addressed, and so it was not clear if the parasite could respond in a way to counteract the effect of the drug. Some of the artesunate-induced changes in gene expression might reflect a protective response by the parasite, since transgenic over-expression of a tryptophan-rich protein unique to *Plasmodium* parasites (previously identified as over-expressed in response to artesunate [4]) reduces artesunate sensitivity [13].

ART therapy is used to treat *P. falciparum* and *P. vivax* infections, and various ART compounds including dihydroartemisinin (DHA) are similarly potent against rodent malaria parasite species such as *P. berghei* [14]. Given the similarity of drug action against different *Plasmodium* species, it is plausible that there is a common pattern of transcriptional response against this class of drug. In this study, we explored *Plasmodium* response to ART with the aim of identifying patterns of gene expression across developmental stages and species that could give further insight into mechanism of drug action. We performed microarray analysis of the *P. falciparum* trophozoite DHA response, and performed a meta-analysis of these data together with published microarray data for artesunate and artemisinin responses. The profile of *Plasmodium* response to DHA was explored further by RNA-seq analysis of drug-treated synchronized *P. falciparum* ring, trophozoite and schizont cultures and mixed stage *P. berghei* in infected mice.

## Results

### *P. falciparum* K1 strain response to DHA assessed by microarray analysis

We tested the transcriptional response to DHA, as this compound is an active metabolite of ART compounds in clinical use. The transcriptional response to this compound was tested for *in vitro* treatment of *P. falciparum* K1 using an experimental design similar to that used previously for artesunate [4]. Synchronized trophozoite cultures were exposed to therapeutic concentrations of DHA (500 nM) for 1, 2 and 3 h. The transcriptomic changes induced by the drug were determined by comparing microarray profiles of drug treated with matched vehicle-treated controls. A major transcriptional response was observed after 1 h, as revealed by the 1886 features showing significant differential expression. After removal of dubious and multiple features mapping to the same gene, 1653 differentially expressed genes were identified. Similar numbers of significant features were found for the other treatment time-points (Additional file 1).

**Fig. 1:**
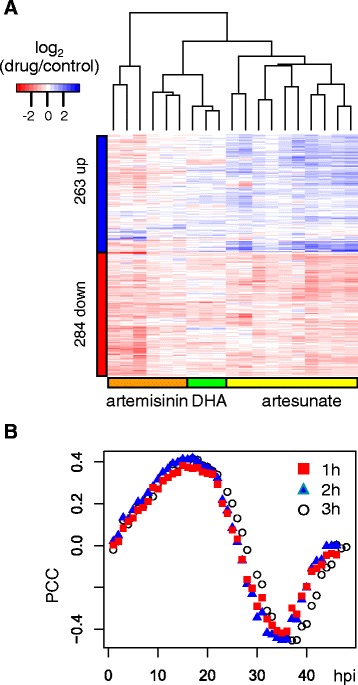
Artemisinin and derivative drug (ART) response from microarray data analysis. **a**. Heatmap of genes with significant changes in expression (547 genes FDR < 0.05) by RankProducts meta-analysis of microarray data for artemisinin [5], artesunate [4] and DHA (this study) induced changes. Rows correspond to genes and columns parasite drug treatments. The values used for clustering are the average of treatment replicates, or the average of multiple probe measurements for the same gene (Hu et al. data [5]). Columns and rows were ordered by hierarchical clustering; the row dendrogram is omitted for clarity. Treatments cluster according to drug derivative/microarray study, as shown by side bars. **b**. Correlation of DHA-induced transcriptional changes (1, 2 and 3 h treatments) in *P. falciparum* K1 strain with HB3 strain intra-erythrocytic developmental cycle changes over 46 time-points of hours post-invasion (hpi) [31]. Pearson correlation coefficients (PCC) were calculated from 4157 shared oligo probe features

The DHA responses were compared with those reported for artesunate [4] and artemisinin [5]. On the one hand, we were not expecting much correspondence among these datasets since different compounds at different concentrations and treatment regimens were used, parasite strains were different, and microarraydesigns and protocols differed. On the other hand, a reproducible set of genes may be found if drugs with the same pharmacophore provoke a similar response. From meta-analysis of expression changes across different treatments, a set of 284 down- and 263 up-regulated significant genes were found (Additional file 2: Fig. 1a). Gene ontology (GO) analysis showed significant enrichment of the terms GO:0043228 ~ non-membrane-bounded organelle and GO:0043232 ~ intracellular non-membrane-bounded organelle among down-regulated genes (FDR = 4.5 %), which primarily relate to the cytoplasmic ribosome. No terms were significant among up-regulated genes. The DHA-induced changes positively correlated with changes observed during normal ring and early trophozoite development (0–28 h post infection (hpi)), whereas drug-induced changes negatively correlated with mature trophozoite and schizont stages (30–48 hpi) (Fig. 1b).

### *Plasmodium falciparum* DHA response assessed by RNA-seq data analysis

**Fig. 2:**
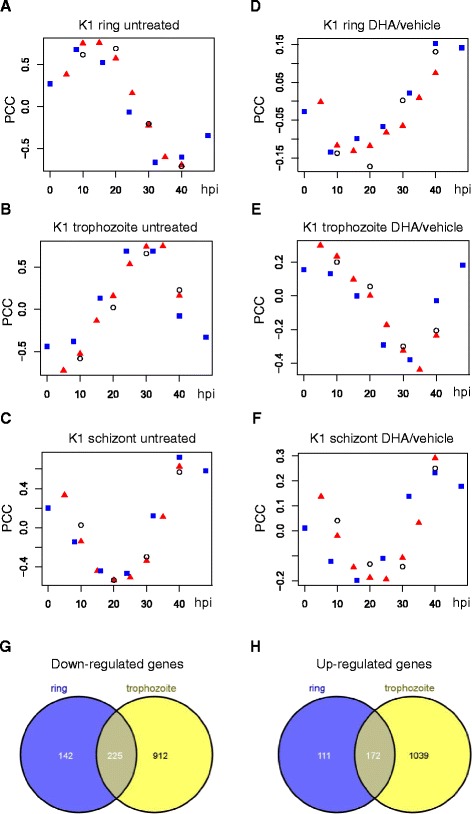
Correlation of DHA drug-induced and developmental transcriptional changes from RNA-seq data. RNA-seq data from untreated *P. falciparum* K1 ring (**a**), trophozoite (**b**) and schizont (**c**) synchronized parasites were compared with published RNA-seq data from time-points taken during the intra-erythrocytic developmental cycle (IDC) (Bártfai et al. dataset [58], red triangles; Otto et al. dataset [57], blue squares; Siegel et al. dataset [59], open circles). Pearson correlation coefficients (PCC) were calculated from 3839 genes expressed in all time-points, defined as hours post-invasion (hpi). Transcriptomic changes in expression induced by 1 h DHA treatment for ring (**d**), trophozoite (**e**) and schizont (**f**) parasites were correlated with changes during the IDC (data and symbols as in parts A-C). The overlap of genes with significant change in expression under DHA treatment in ring and trophozoite parasites is shown for down-regulated genes in (**g**) and up-regulated genes in (**h**)

*In vitro* DHA treatments of *P. falciparum* K1 were repeated (500 nM DHA for 1 h), but this time on synchronized ring, trophozoite and schizont parasites. The transcriptomes of the untreated parasite populations showed maximal correlation with early (10–20 hpi), mature (24–35 hpi) and late (40–48 hpi) time-points of the IDC from published RNA-seq studies, respectively (Fig. 2a, b, c). Drug treatments had no effect on parasite morphology (Additional file 3). The transcriptional responses to DHA among the different stage parasites were determined by comparing strand-specific RNA-seq read counts of drug-treated with matched vehicle-treated controls.

The global profile of transcriptomic changes induced by DHA for rings and trophozoites were almost mirror-image profiles of untreated parasites at the same stages of development (Fig. 2d, e). In contrast, the patterns of gene expression in DHA-treated schizonts were indistinguishable from normal schizont development (Fig. 2f). Of the three stages, the most dramatic response was observed for the trophozoite stage in which 2349 genes showed significant change. In contrast, 651 genes were differentially expressed in ring, and none in schizont stage parasites (Additional file 4). Among the differentially expressed genes in rings and trophozoites, there was considerable overlap (Fig. 2g, h). GO analysis showed that cytosolic ribosome function was strongly represented among down-regulated genes in rings (Table 1) and trophozoites (Table 2). Significant GO terms relating to apicoplast and protein phosphorylation functions were represented among up-regulated genes in trophozoites (Table 3).

**Table 1:** Gene Ontology analysis of genes down-regulated in response to DHA in *P. falciparum* K1 rings from RNA-seq analysis

Term	Count	List Total	Pop Hits	Pop Total	PValue	FDR
GO:0006412 ~ translation	70	151	168	1076	1.5E–22	2.0E–19
GO:0044445 ~ cytosolic part	38	115	61	869	3.8E–20	4.3E–17
GO:0005840 ~ ribosome	48	115	108	869	8.3E–18	9.2E–15
GO:0005829 ~ cytosol	42	115	84	869	1.3E–17	1.4E–14
GO:0003735 ~ structural constituent of ribosome	44	184	96	1289	2.4E–14	3.0E–11
GO:0030529 ~ ribonucleoprotein complex	52	115	150	869	7.4E–14	8.2E–11
GO:0005198 ~ structural molecule activity	44	184	115	1289	4.8E–11	6.1E–08
GO:0043228 ~ non-membrane-bounded organelle	49	115	167	869	8.7E–10	9.6E–07
GO:0022627 ~ cytosolic small ribosomal subunit	16	115	22	869	1.9E–09	2.1E–06
GO:0015935 ~ small ribosomal subunit	19	115	35	869	2.5E–08	2.8E–05
GO:0008135 ~ translation factor activity, nucleic acid binding	17	184	47	1289	3.8E–04	4.8E–01
GO:0006006 ~ glucose metabolic process	10	151	20	1076	6.7E–04	9.1E–01
GO:0016052 ~ carbohydrate catabolic process	10	151	21	1076	1.0E–03	1.4E + 00
GO:0044275 ~ cellular carbohydrate catabolic process	10	151	21	1076	1.0E–03	1.4E + 00
GO:0046164 ~ alcohol catabolic process	10	151	21	1076	1.0E–03	1.4E + 00
GO:0005853 ~ eukaryotic translation elongation factor 1 complex	5	115	5	869	1.3E–03	1.4E + 00
GO:0019318 ~ hexose metabolic process	10	151	22	1076	1.6E–03	2.1E + 00

Enrichment of GO terms in 367 significantly down-regulated genes (gene list) was calculated relative to the total of 4075 genes detected as expressed in this stage (background list). The GO terms listed are those considered significant from DAVID analysis (FDR < 5 %) and non-redundant by REVIGO filtering

**Table 2:** Gene Ontology analysis of genes down-regulated in response to DHA in *P. falciparum* K1 trophozoites from RNA-seq analysis

Term	Count	List Total	Pop Hits	Pop Total	PValue	FDR
GO:0005829 ~ cytosol	70	276	85	963	3.4E–26	4.2E–23
GO:0044445 ~ cytosolic part	54	276	62	963	4.6E–22	5.8E–19
GO:0006412 ~ translation	99	348	181	1139	3.1E–13	4.5E–10
GO:0005840 ~ ribosome	68	276	117	963	3.1E–12	3.8E–09
GO:0003735 ~ structural constituent of ribosome	62	407	101	1385	1.4E–11	2.0E–08
GO:0022627 ~ cytosolic small ribosomal subunit	22	276	22	963	3.5E–11	4.3E–08
GO:0030529 ~ ribonucleoprotein complex	82	276	159	963	4.5E–11	5.6E–08
GO:0043228 ~ non-membrane-bounded organelle	87	276	186	963	7.1E–09	8.9E–06
GO:0005198 ~ structural molecule activity	65	407	120	1385	6.6E–09	9.3E–06
GO:0015935 ~ small ribosomal subunit	25	276	35	963	6.5E–07	8.1E–04
GO:0000502 ~ proteasome complex	20	276	30	963	5.9E–05	7.4E–02
GO:0044265 ~ cellular macromolecule catabolic process	29	348	55	1139	9.2E–04	1.3E + 00

Enrichment of GO terms in 1137 significantly down-regulated genes (gene list) was calculated relative to the total of 4636 genes detected as expressed in this stage (background list). The GO terms listed are those considered significant from DAVID analysis (FDR < 5 %) and non-redundant by REVIGO filtering

**Table 3:** Gene Ontology analysis of genes up-regulated in response to DHA in *P. falciparum* K1 trophozoites from RNA-seq analysis

Term	Count	List Total	Pop Hits	Pop Total	PValue	FDR
GO:0009536 ~ plastid	122	233	405	963	2.5E–04	0.31
GO:0006468 ~ protein amino acid phosphorylation	25	282	51	1139	3.1E–04	0.45
GO:0020011 ~ apicoplast	121	233	403	963	3.3E–04	0.41
GO:0006793 ~ phosphorus metabolic process	35	282	85	1139	8.4E–04	1.20
GO:0006796 ~ phosphate metabolic process	35	282	85	1139	8.4E–04	1.20
GO:0004674 ~ protein serine/threonine kinase activity	22	353	44	1385	9.6E–04	1.31
GO:0016310 ~ phosphorylation	30	282	70	1139	1.1E–03	1.52

Enrichment of GO terms in 1211 significantly up-regulated genes (gene list) was calculated relative to the total of 4636 genes detected as expressed in this stage (background list). The GO terms listed are those considered significant from DAVID analysis (FDR < 5 %) and non-redundant by REVIGO filtering

**Fig. 3:**
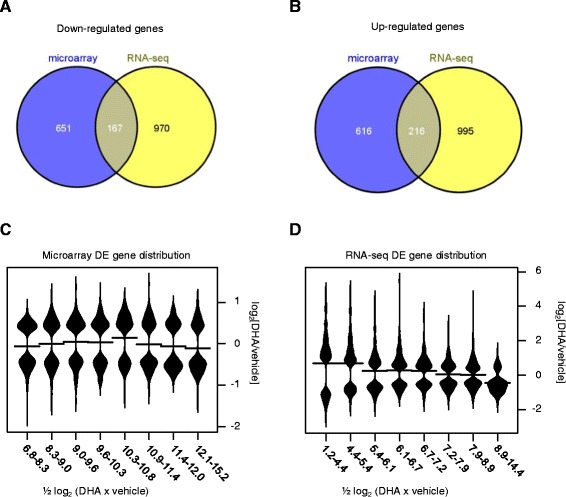
Comparison of *P. falciparum* K1 DHA-responsive genes from microarray and RNA-seq data. The genes identified as showing significant changes in expression in response to 1 h DHA treatment for *P. falciparum* K1 trophozoites from microarray (1653 genes) and RNA-seq (2349 genes) experiments were compared. The overlap of down-regulated genes is shown in (**a**) and overlap of up-regulated genes in (**b**). The distributions of DHA-induced change in expression of differentially expressed genes with respect to average level of gene expression are shown for microarray in (**c**) and RNA-seq in (**d**). The log_2_ (DHA/vehicle) values for 1653 genes from microarray and 2349 genes from RNA-seq were divided equally into 8 intervals of average expression. The distributions of log_2_ (DHA/vehicle) values in each interval were determined by beanplot analysis; the mean gene expression change in each interval is shown by a horizontal line and the distributions of gene expression change are indicated by the “bean” shapes. Wilcoxon rank sum test of these distributions showed that 3/28 pairwise comparisons in the microarray data are significant (p < 0.05), whereas 21/28 pairwise comparisons in the RNA-seq data are significant (*p* < 0.05)

RNA-seq is a recent, maturing technology which has not yet been used extensively to study transcriptomic responses to drugs. We compared data obtained for the *P. falciparum* K1 strain trophozoite response from the two transcriptomic approaches. Although many differentially expressed genes were called as significant, the overlap between microarray and RNA-seq was rather low, with only 167 down- and 216 up-regulated genes in common (Fig. 3a, b). To investigate the possible reasons for the discordance between experimental approaches, the distributions of up- and down-regulated genes with respect to the average expression level were determined. For microarray significant genes, the distribution of up-and down-regulated genes is about the same irrespective of the gene expression level (Fig. 3c). In contrast for RNA-seq, a bias towards up-regulation is observed for lowest-expressed genes and a down-regulation bias is apparent for highest expressed genes (Fig. 3d).

### *Plasmodium berghei in vivo* DHA response assessed by RNA-seq data analysis

**Fig. 4:**
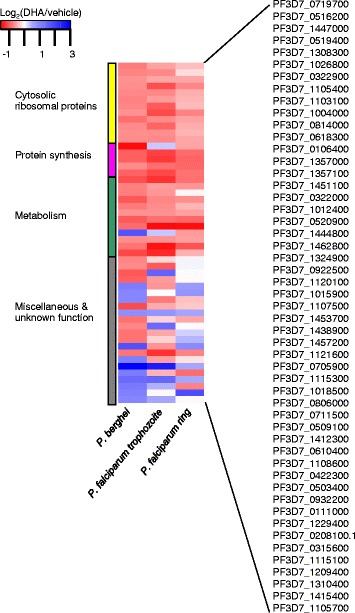
DHA-responsive genes conserved among *Plasmodium* species. The heatmap shows average DHA-induced changes relative to vehicle control from two replicates, as determined by limma analysis of RNA-seq read count data. 51 genes orthologous between *P. berghei* ANKA and *P. falciparum* K1 showed significant change in response to DHA in both species. The ordering of genes in rows and columns is arbitrary. Genes with related functions are grouped, as shown by the side-bars. Genes are labeled by the *P. falciparum* orthologue gene ID

Next, we tested whether the transcriptomic response to DHA treatment is conserved among *Plasmodium* parasite species with similar sensitivity to the drug. Drug treatments were performed on *P. berghei in vivo* in infected mice. Although *P. berghei* infections are not synchronous, parasites in the peripheral animal blood are mostly ring and trophozoite stages. Given the high degree of overlap in genes showing a significant change in expression in response to DHA between synchronous ring and trophozoite *P. falciparum* (Fig. 2g, h; Tables 1, 2, 3), we reasoned that we would find a similar pattern in terms of DHA-responsive gene functions in the mixed ring/trophozoite stage *P. berghei*. The lack of synchronicity and a suitable dataset for comparing with changes throughout the IDC prevented us from determining whether *P. berghei* also shows a global arrest response in ring and trophozoite stages. The strand-specific RNA-seq data from DHA-treated parasites were compared with vehicle-treated controls. 87 genes showed significant changes in expression (Additional file 5). Some of these genes have no orthologues in *P. falciparum*, including 14 annotated as exported proteins specific to rodent parasite species. Of the 68 genes remaining that are orthologous in *P. falciparum*, 51 *P. falciparum* orthologues also showed significant change in response to DHA in ring and/or trophozoites (Fig. 4). The majority of these DHA-responsive genes common between species were down-regulated. Moreover, cytosolic ribosome function was significantly represented among these genes by GO analysis (Table 4).

**Table 4:** Gene Ontology analysis of genes differentially expressed in response to DHA in *P. berghei* ANKA from RNA-seq analysis

Term	Count	List Total	Pop Hits	Pop Total	PValue	FDR
GO:0043228 ~ non-membrane-bounded organelle	15	21	178	856	2.2E–06	0.002
GO:0043232 ~ intracellular non-membrane-bounded organelle	15	21	178	856	2.2E–06	0.002
GO:0005198 ~ structural molecule activity	11	30	113	1247	1.2E–04	0.121
GO:0003735 ~ structural constituent of ribosome	10	30	95	1247	1.7E–04	0.174
GO:0044445 ~ cytosolic part	8	21	59	856	2.0E–04	0.177
GO:0005829 ~ cytosol	9	21	82	856	2.5E–04	0.220
GO:0015935 ~ small ribosomal subunit	6	21	34	856	7.4E–04	0.658
GO:0022627 ~ cytosolic small ribosomal subunit	5	21	22	856	1.2E–03	1.078
GO:0006412 ~ translation	12	29	168	1023	2.8E–03	3.199
GO:0006096 ~ glycolysis	4	29	13	1023	4.4E–03	4.910

GO analysis was performed using corresponding *P. falciparum* orthologous genes. Enrichment of GO terms in 68 differentially expressed genes with *P. falciparum* orthologues (gene list) was calculated relative to the total of 4084 genes detected as expressed in the *P. berghei* ANKA samples with *P. falciparum* orthologues (background list). The GO terms listed are those considered significant from DAVID analysis (FDR < 5 %) and non-redundant by REVIGO filtering

## Discussion

*Plasmodium* parasite transcriptional responses to the anti-malarial drug DHA were studied. We performed short treatments of drug at high concentration to mimic what the parasite would have to respond to in a typical therapeutic intervention. The transcriptional response to DHA for the *P. falciparum* K1 strain was first explored by microarray to permit comparison with other drug responses at the same trophozoite stage in this strain. DHA exposure elicits a marked response that is very similar among the three time-points tested (Fig. 1; Additional file 1). The majority of trophozoites are inviable after 1 h exposure to 500 nM DHA [10]. Therefore, the consistent transcriptomic pattern of DHA response in trophozoites is a signature of loss of viability. Moreover, this pattern is consistent across independent microarray studies measuring response to different artemisinin derivatives (Fig. 1a). This loss of viability pattern is obscured in the transcriptomic response to slower acting anti-malarial drugs such as pyronaridine and chloroquine [15] and antifolates [3], perhaps because of confounding transcriptomic changes occurring through normal cell cycle progression during the longer drug treatment period needed to observe a significant transcriptional response.

Correlation analyses of ring and trophozoite DHA treatment profiles with the IDC show that these parasite stages respond to the drug by global reprogramming of transcriptomes. This is shown most markedly for trophozoites by the negative correlation between DHA treatment profiles and mid-cycle IDC time-points corresponding to trophozoite maturation (Figs. 1b and 2e). Apart from a brief early ring phase, this is the same period during the IDC when parasites are most vulnerable to DHA [10, 12]. The transcriptome of rings is similarly reprogrammed in that there is negative correlation of DHA-induced changes with ring and trophozoite time-points during the IDC (Fig. 2d). Moreover, there is extensive overlap of differentially expressed genes induced by DHA between rings and trophozoites (Fig. 2g, h). The ring and trophozoite transcriptional response to DHA may thus be an arrest mechanism to retard metabolic processes essential for maturation, as shown by down-regulation of glycolysis and protein synthesis genes (Tables 1 and 2). In contrast, schizonts do not respond to DHA (Fig. 2f). The lack of transcriptional response in schizonts can be explained by their reduced protein synthesis [16] and transcription [17]. Moreover, schizonts are also less able to down-regulate gene expression because of an overall reduced rate of mRNA decay [18].

The maturation and metabolic status of the cell is critical in determining sensitivity to ART across other cell types, since actively dividing mammalian cells are more susceptible to ART-induced cell death than quiescent cells [19]. The arrest transcriptomic pattern in DHA-treated rings may be a trigger for a physiological protective change, e.g. dormancy. We did not observe dormant parasites in our experiments (Additional file 3) as we harvested parasites immediately after a short treatment period (dormant parasites are observed after a 6–13 h lag [10, 12, 20, 21]). Strains with lower sensitivity to ART are better able to enter, or recover from dormancy [22–24]. A similar trait has been observed in parasite isolates from slow-clearing infections [1, 2] and a rodent malaria model [25], suggesting that *in vitro* tests of ART response correspond well to the situation *in vivo*. In trophozoites, the transcriptional response to DHA does not lead to a protective physiological change, perhaps because parasites at this stage are committed to an anaplerotic metabolic state in which glycolysis, TCA cycling and glutamiolysis occur at high rates [26]. The commitment to anaplerosis may occur quite early in the IDC, since ART-resistant strains are significantly more tolerant of 700 nM DHA than sensitive strains only for early ring stages of development [2, 12]. Recently, it has been shown that ART-resistant clones from slow-clearing infection isolates have elongated ring and shortened trophozoite development stages [21]. These adaptations enhance the parasite’s ability to become a dormant ring and minimize the ART-vulnerable trophozoite stage.

The arrest response to ART in *P. falciparum* rings and trophozoites, perhaps acting as a trigger to prevent the parasite from maturing, implies that key metabolic functions (not yet identified) may be disrupted by ART. *Plasmodium* is exquisitely sensitive to ART, in part because of oxidative damage mediated by interaction of ART with heme, a major source of molecular iron. Although hemoglobin-heme is highly abundant in the parasite’s food vacuole, this may not be the major site for the drug’s killing effect since the ingested content containing red cell cytosolic proteins such as haemoglobin and catalase can help to detoxify artemisinin [27]. Another site of parasite heme and molecular iron is the mitochondrion, in which these cofactors are essential for the functions of cytochromes and iron-sulfur proteins. Although some genes with mitochondrial function are differentially expressed in response to DHA, e.g. up-regulation of PF3D7_0915000 (*ndh2*) and PF3D7_1034400 (*sdha*) genes in trophozoites (Additional file 4), there is no concerted regulation of genes with mitochondrial function as shown by the absence of related significant GO terms (Tables 1–3). The lack of a coordinated parasite response regarding mitochondrial function suggests that late rings and trophozoites are committed to mitochondrial biogenesis, which is perhaps linked to commitment to anaplerosis (see above). Maturing parasites committed in this manner are vulnerable to ART because mitochondrial biogenesis exposes the cell to the oxidative stress effects of ART acting in combination with heme and molecular iron. The inability of committed cells to arrest mitochondrial biogenesis imposes a futile energy expenditure, since mitochondrial function is ablated by ART (shown by rapid loss of mitochondrial membrane potential following ART treatment [28, 29]). Merozoites are less vulnerable to ART [11], because they are equipped with poorly developed mitochondria and have low metabolic requirements. Schizonts have low metabolic requirement and are less sensitive to ART than trophozoites as they have fully developed mitochondria. We therefore postulate that maturing parasites committed to mitochondrial biogenesis are fated to ART-induced death, whereas young uncommitted cells can become dormant. Our data are thus consistent with the proposal in [30] that *Plasmodium* maintains expression of most mitochondrial genes, which enables rapid recovery of mitochondrial function when exiting from ART-induced dormancy.

Among genes showing significant down-regulation in response to DHA in *Plasmodium* rings and trophozoites, it is notable that cytosolic ribosome function is represented among significant GO terms from both microarray and RNA-seq data (Tables 1, 2 and 4). This coordinated down-regulation is particularly striking for DHA-treated rings, since expression of these genes is maximal during the first 20 h of normal development [31]. The coordinated down-regulation of *Plasmodium* ribosomal protein genes is not surprising since they possess a common regulatory element in their promoters, the G-box, and putative positive and negative transcription factors to control their expressions have been described [32]. It is interesting to note that concerted repression of yeast cytosolic ribosomal protein genes also occurs under hydrogen peroxide stress, and this response is dependent on thiol peroxidase enzymes [33]. *Plasmodium* lack catalase and GPx enzymes, and so they rely on thiol peroxidase enzymes and the NADPH pathway for maintaining redox balance [34]. It is tempting to speculate that thiol peroxidase enzymes become oxidized under ART stress similar to hydrogen peroxide stress. The accumulation of these oxidized enzymes could trigger a peroxide-signalling event [35] that activates the observed gross transcriptional response in parasites. One potential regulator of such a peroxide-signalling event is the nuclear thiol peroxidase PfnPrx, which is essential and has intimate association with most of the parasite genome [36].

Of the significant GO terms among up-regulated trophozoite genes in response to DHA (Table 3), apicoplast function was recently shown by qRT-PCR to be up-regulated in *P. falciparum* recovering from DHA treatment; the up-regulation of pyruvate metabolism in the apicoplast is thought to compensate for reduced ATP levels caused by down-regulation of glycolysis and TCA cycle [30]. Among the up-regulated genes representing significant protein phosphorylation GO terms are 18 FIKK kinase genes (Additional file 4). FIKK kinases are exported to the host red cell and play important roles in pathogenesis [37, 38]. In accordance with the findings made by Natalang et al. for artesunate response [4], many other genes for exported proteins from different families such as hyp, PHIST, Pfmc-2TM, PfEMP1, RESA, ETRAMP, SURFIN are significantly up-regulated in DHA-treated *P. falciparum* trophozoites (Additional file 4). The expressions of *Plasmodium* translocon of exported proteins (PTEX) genes recently shown to be essential for protein export [39, 40] are also significantly up-regulated in DHA-treated trophozoites (Additional file 4). Increased protein export may be the final act of dying parasites to restore the energy deficit, since exported proteins are essential for uptake of nutrients [40].

Recently, mutations in the PF3D7_1343700 gene encoding K13 protein have been associated with artemisinin resistance in Southeast Asia [41]. Interestingly, this gene is significantly up-regulated in response to DHA in trophozoites (Additional files 1 and 4). K13 mutations in ART resistant parasites appear to reduce ubiquitinylation of proteins, including PfP13K [42]. The expression of the PfPI3K encoding gene PF3D7_0515300 does not change in response to DHA (Additional files 1 and 4). The up-regulation of K13 in response to DHA could reduce PfP13K protein through the ubiquitin/proteasome pathway, and in turn lead to reduced phosphatidyl inositol-3-phosphate (PI(3)P). Reduced PI(3)P could lead to interference of protein export from the endoplasmic reticulum (ER), which is dependent on PI(3)P [43]. Interference of protein export would presumably lead to ER stress, for which *P. falciparum* is notably vulnerable [44]. Among the genes with altered expression during normal development in ART resistant parasites, genes encoding proteins in the unfolded protein response (UPR) are notably up-regulated [7]. The key genes in this pathway, i.e. PF3D7_1108600 (ERC), PF3D7_0917900 (HSP70-2 or BiP), PF3D7_0322000 (CYP19A), PF3D7_1357800 (TCP-1/cpn60), PF3D7_0306800 (TCP1-b) and PF3D7_0718500 (prefoldin) are significantly down-regulated in *P. falciparum* ring and/or trophozoites exposed to DHA (Additional file 4). Furthermore, the *P. berghei* orthologues of CYP19A and ERC (PBANKA_121650 and PBANKA_093840, respectively) are also significantly down-regulated in response to DHA (Additional file 5). From these observations, it appears that ART resistant parasites have adaptations in the arrest transcriptional response to specifically counteract the ER stress induced by ART, in particular reversal of expression changes that could actually sensitize the parasite to drug. This trend is also apparent for genes with antioxidant function, which are up-regulated in DHA-resistant parasites to counteract the oxidant stress induced by the drug [9]. Among genes with known antioxidant function, the PF3D7_1438900 (thioredoxin peroxidase1), PF3D7_1457200 (thioredoxin 1), and PF3D7_1121600 (EXP1, an exported glutathione transferase which mediates sensitivity to artesunate [45]) genes are down-regulated in response to DHA in *P. falciparum* trophozoites (Additional file 4) and the orthologues in *P. berghei* (Additional file 5).

The *in vivo* DHA response in *P. berghei* was strikingly similar to the ring and trophozoite responses in *P. falciparum*, as shown by the high correspondence of differentially expressed orthologous genes from RNA-seq data (Fig. 4) and with the same significant representation of cytosolic ribosome function by GO analysis (Table 4). The transcriptional responses between species were similar despite the fact that the *P. berghei* were not synchronous and the read depth was much lower for *P. berghei* owing to contaminating mouse mRNA, as shown by the high percentage of reads mapping to *Mus musculus* (Additional file 6). This could mean that the transcriptional response in ring and trophozoite stages to DHA (see above) is conserved among *Plasmodium* parasite species with similar sensitivity to the drug, although this would need to be investigated further with synchronous parasites.

Although similar overall patterns of DHA-response in *P. falciparum* trophozoites were found among microarray and RNA-seq data, there was rather poor agreement in terms of specific genes showing significant changes in expression (Fig. 3). The *P. falciparum* microarray data analyzed in this study were generated from two-channel platforms. These data must be normalized using aggressive techniques such as locally weighted linear regression to correct for intensity-dependent dye bias due to differences in physical properties of the two cyanine fluorescent dyes. Data normalization in this fashion can be inaccurate though when central assumptions are violated, for example when a high proportion of genes are differentially expressed, and/or there is a skewed direction of change in gene expression. In this situation, external RNA controls may be required [46, 47]. This approach is rarely used though as it depends crucially on the quality of the external controls, and appropriate probes for the controls designed on the array. Normalization of RNA-seq data is simpler than microarray as it involves re-scaling of global read counts. In some cases where mRNA content varies greatly among cell types, external controls may be required for normalization, as proposed in microarray experiments [48]. However, the standard Trimmed Mean of M-values (TMM) normalization method, as we have used in our RNA-seq experiments, is robust to skewed changes provided mRNA contents are similar [49]. For the DHA-responsive genes found by RNA-seq, we found a clear bias in that highly expressed genes were skewed towards down-regulation, and the opposite skew for low-expressed genes. In contrast, there was no marked bias from normalized microarray data (Fig. 3). We think that the microarray normalization procedure removes the skew, which leads to inaccurate normalization, and consequently inaccuracy in identification of differentially expressed genes. In contrast, RNA-seq data normalization is more accurate, as shown by greater statistical support for differentially expressed genes and high reproducibility, even among different species.

## Conclusions

Our data show that RNA-seq is a powerful tool for identifying the malaria parasite’s response to anti-malarial drugs. In particular, the arrest response at ring and trophozoite stages to DHA is clearly evident from RNA-seq data, but is less obvious from microarray data. As sequencing costs continue to fall, a far more detailed study than we have done of the transcriptional response to anti-malarial drug could be undertaken. In particular, more extensive sampling of drug treatment including parasite starting age, drug dose and time-point of treatment may reveal the full complexity of the arrest response employed by the parasite. This knowledge could lead to more effective use of existing drugs as we would be able to deploy drugs in combinations and therapies to circumvent the protective response by the parasite, and possibly forestall drug resistance.

## Methods

### Ethics statement

This study was approved by the BIOTEC committee for use and care of laboratory animals.

### *P. falciparum* K1 parasite culture and microarray

*P. falciparum* K1 strain was cultured *in vitro* according to standard procedures [15, 50]. Parasites cultures were synchronized to ring stages by two consecutive sorbitol treatment cycles [51]. 48 h after the second synchronization, the parasite culture was divided equally into new culture plates for drug treatment. The synchronized parasites were then cultured for 18 h to obtain a majority of trophozoite-stage parasites for drug treatment. Parasites were treated with 500 nM DHA (Dafra Pharma, Belgium) or vehicle solvent control (0.1 % (v/v) DMSO). Parallel culture plates were left untreated as reference controls. Samples were taken after 1, 2, and 3 h of treatment. Parasites were harvested from culture and liberated from host cells by treatment with 0.15 % (w/v) saponin for 5 min on ice. The liberated parasites were washed three times with 1xPBS (137 mM NaCl, 8 mM KCl, 10 mM Na_2_HPO_4_, 2 mM KH_2_PO_4_, pH 7.4) to remove host cell debris and drug.

Total RNA was extracted from parasites using Trizol reagent (Invitrogen) following the manufacturers’ recommendations. Genomic DNA was removed from total RNA using a Turbo DNA free™ kit (Ambion, Applied Biosystems). The RNA was reverse-transcribed to cDNA using oligo-dT_(21)_ primer, amino-allyl dUTP and ImpromII enzyme (Promega), and fluorescent cyanine dye coupled to amino-allyl labeled cDNA as described in [52]. cDNA from DHA or vehicle treated parasites was labeled with Cy5 and culture-matched control untreated parasite cDNA labeled with Cy3. Approx. 5 pmol of each Cy-dye labeled cDNA (estimated by NanoDrop ND1000) was applied to each DNA microarray. The microarray platform containing 8088 70-mer oligo probes was described previously in [15]. Five independent culture replicate experiments were conducted. Microarray hybridization, washing and scanning were performed as described in [15]. The images of scanned microarrays were analyzed using the GenePix Pro 6.1.0.4 package. Microarray feature signals of irregular shape or with obvious defects, e.g. large dust specks or scratches were flagged (i.e. marked as not available for analysis) manually in the GenePix software and raw data saved in .gpr file format. A total of 29 microarray hybridizations were performed [53].

### RNA-seq library construction

*P. falciparum* K1 strain was cultured *in vitro* and synchronized to ring stages as described above. Parasites were treated with 500 nM DHA or vehicle solvent control (0.1 % DMSO), immediately, 18 or 36 h after the second synchronization, corresponding to ring, trophozoite or schizont populations, respectively. Parallel plates of parasites from the same synchronized culture were left untreated as controls. Parasites were harvested from culture after 1 h of treatment and processed immediately for RNA extraction.

*Plasmodium berghei* (ANKA) 507m6cl1 MRA-867 was obtained through the MR4 as part of the BEI Resources Repository, NIAID, NIH, deposited by C.J. Janse and A.P. Waters. 8-week old female ICR mice (approx. 30 g each) were infected with 1 × 10^7^*P. berghei* parasites each and 24 h post-infection, animals were injected i.p. with DHA (10 mg/kg) or an equal volume of vehicle control (30 % DMSO in distilled water). Parasites were obtained by cardiac puncture 2 h post-injection of drug or vehicle and harvested in heparinized tubes. White cells were separated from infected red cells using home-made CF-11 columns (approx. 4 ml packed resin). The parasitized red cells were then collected by centrifugation from the filtered blood. Harvested *P. falciparum* and *P. berghei* were liberated from host cells, total RNA extracted, and genomic DNA removed as described above.

0.7–4 μg of total RNA from each treatment condition was converted to a strand-specific RNA-seq sequencing library using a TruSeq Stranded mRNA LT Sample Prep kit following the manufacturers’ recommendations (Illumina). A different TruSeq adapter was used for each RNA-seq library to allow pooling of samples for Illumina sequencing on the same flow cell. Each experiment was performed in duplicate with an independent parasite culture (*P. falciparum*), or different infected host animal (*P. berghei*). The RNA-seq libraries were quantified by QuantIT dsDNA High Sensitivity kit (Invitrogen) and by qPCR using a library quantification kit (Kapa Biosystems). Libraries were pooled in equimolar ratios and applied to a MiSeq flow cell at 12 pM with 1 % phiX174 control library (Illumina). Paired-end sequencing was performed on two MiSeq v2 2x150 bp flow cells according to the manufacturers’ recommendations (Illumina). The raw data from the MiSeq instrument were de-multiplexed and fastq files generated for each RNA-seq library [53].

### Microarray data analysis

Statistical analysis of microarray and RNA-seq data for differential gene expression was performed using the limma package [54] implemented in R 3.0.2. For analysis of microarray data from DHA experiments, the .gpr microarray files were used as input. Microarray features flagged by the Genepix software, designated as empty (no oligo probe), not in genome, negative control, positive control, plastid genome, unmapped, or mapping to non-mRNA were given zero weight for normalization using the modifyWeights function in limma. Microarray feature median foreground signals of Cy5/Cy3 ratios were background corrected with normexp.offset = 50, and normalized within microarrays by global loess. Statistical analysis of differences in normalized Cy5/Cy3 ratio was performed using limma’s linear model fitting comparing normalized signals of DHA treated/untreated as one group with vehicle treated/untreated as another group. Model fitting was performed using feature weights within each array and across arrays using array weights. Features with Benjamini-Hochberg adjusted *p*-value <0.05 were considered as significant. For analysis of annotated genes from microarray features called significant, we removed all dubious features that corresponded to defunct gene models, or conflicted in terms of direction of change for multiple features belonging to the same gene model.

Microarray data meta-analysis was performed as follows. The average log2 normalized values of DHA/vehicle induced change from limma output were obtained for all features. The published average normalized values for artesunate versus untreated parasites were obtained from Natalang et al. Additional file 2 in [4]. From these normalized values, the feature with the lowest reported *p*-value from limma statistical testing of differential expression was taken to represent the annotated gene to which that probe is designed against. For artemisinin, the published normalized log2 Cy5/Cy3 values from control treatments were subtracted from values of corresponding artemisinin time-points as available from Hu et al. Supplementary Table 2 in [5]. A data input file was constructed for meta-analysis containing values for 3196 genes, in which columns contained average normalized values of drug versus control treatment for different treatment time-points. Statistical testing of differential gene expression across the independent datasets was performed using the RPadvance package from the RankProduct suite [55] in R.3.0.2. Genes with pfp <0.05 were considered as significant.

Correlation of DHA-induced transcriptomic changes with those during normal intra-erythrocytic development (IDC) was performed as follows. The average normalized fold-change data across 46 IDC time-points for *P. falciparum* strain HB3 were obtained from the Bozdech et al. dataset S2 in [31]. These values were log2 transformed and a data file constructed containing these values and the average log2 normalized DHA/vehicle values from limma output for the same features (same oligonucleotide probe ID). Features with missing values were removed leaving 4157 for Pearson correlation analysis.

### RNA-seq data analysis

The raw reads from fastq files were first filtered to remove matches to rRNA by BLAST alignment. Pre-processing was performed by trimming low-quality base-calls (N) from reads, followed by removal of exact duplicates and reads of low overall quality. The pre-processed reads were mapped to the *P. falciparum* 3D7 2013-3-1 reference genome, or *P. berghei* ANKA 2013-3-1 and *Mus musculus* build 38 reference genomes (*P. berghei* experiments) using Bowtie2 and TopHat2 programs. Potential duplicate and inaccurately mapped reads were removed, and gene expression counts were determined from counts of sense-strand reads (read 1 only) mapping within (complete and partial overlap) annotated genes. The RNA-seq data processing results are shown in Additional file 6. The full details of the RNA-seq data processing, including custom perl scripts are provided in Additional file 7.

The read counts from mapped RNA-seq data were used as input for limma analysis. Read count data were filtered to remove non-protein coding genes and genes with read counts per million <1 in one or more library. The read counts were normalized using the trimmed mean of M values function implemented in the edgeR package [49]. The normalized read count data were then used to calculate mean-variance relationships and sample weights in voom [56]. Tests for differentially expressed genes were performed using the linear model of DHA versus vehicle treatment in limma [54]. Genes with Benjamini-Hochberg adjusted *p*-value <0.05 were considered as significant.

Correlation analysis of IDC changes with DHA treatment from RNA-seq data was performed in a similar fashion to microarray as follows. The published RNA-seq data from IDC time-points [57–59] were obtained from GEO (Bartfai et al. dataset accession number GSE23865) and EMBL Bank (ERP000069, Otto et al. dataset; and ERP001849, Siegel et al. dataset). The raw fastq files were processed and mapped to the *P. falciparum* 3D7 reference genome as described above. The mapped reads were then used to calculate read counts for annotated genes. All reads mapping to annotated genes in the Otto et al. and Bartfai et al. datasets were assumed to originate from the annotated sense mRNA, whereas only the mapped sense-strand reads from total RNA libraries (libraries #1–4) were used for the Siegel et al. dataset. The sum of read counts from all IDC time-points in each dataset was used as a reference for determining changes in gene expression throughout the IDC. The edgeR package [49] implemented in R.3.0.2 was used to calculate log2 changes in gene expression across IDC time-points, in which read counts were normalized in each RNA-seq library using the trimmed-mean of M-values function in edgeR. The average log2 DHA induced changes from RNA-seq calculated by limma (see above) were correlated with log2 IDC changes for genes expressed at read counts per million >1 in all datasets by Pearson correlation.

### Other data analyses

Venn diagrams were made using the VENNY web tool [60]. Gene ontology analysis was performed using the database for annotation, visualization, and integrated discovery (DAVID) web tool [61] with default parameters. The background gene lists comprised all genes with expression values in each dataset. GO terms with FDR <5 % were considered as significant. Redundant GO terms from significant term lists were removed using the REVIGO tool [62]. Heatmaps and beanplots were constructed using the R packages heatmap.2 and beanplot, respectively in R.3.0.2 [63]. Differences in distributions of differentially expressed genes among ranges of average gene expression were tested using the Wilcoxon rank sum with Bonferroni post-hoc test in R.3.0.2 [63].

## Availability of supporting data

The data sets supporting the results of this article are available in the NCBI Gene Expression Omnibus repository, under SuperSeries accession number GSE62136 [http://www.ncbi.nlm.nih.gov/geo/query/acc.cgi?acc=GSE62136].

## Additional files

Additional file 1:
**Microarray data analysis from DHA treatment.** Limma results from 1, 2, and 3 h 500 nM DHA treatments in *P. falciparum* K1 trophozoites. (XLS 2986 kb) Additional file 2:
**Microarray** **meta-analysis** **of ART treatments.** Data input file and RPadvance meta-analysis of microarray data from DHA (this study), artemisinin [5] and artesunate [4] treatments of *P. falciparum* trophozoites. (XLS 1911 kb) Additional file 3:
**Morphology of treated parasites.** Microscopic images were obtained from Giemsa-stained smears of *P. falciparum* synchronized cultures. Scale bars, 5 microns. Parasite counts of each morphologically identifiable stage (ring, troph, schizont) were determined from each experimental condition at harvest after the 1 h treatment period. (PPT 3068 kb) Additional file 4:
**RNA-seq data analysis from DHA treatment of** ***P. falciparum*** **Limma results from 1 h treatments with 500 nM DHA in** ***P. falciparum*** **K1 rings, trophozoites and schizonts.** (XLS 2040 kb) Additional file 5:
**RNA-seq data analysis from DHA treatment of** ***P. berghei.*** Limma results from *in vivo* 2 h treatments of mice infected *P. berghei* ANKA and injected with 10 mg/kg DHA or vehicle control. (XLS 809 kb) Additional file 6:
**Summary of** **RNA-seq** **data preprocessing and read mapping to reference genomes.** (XLS 7464 kb) Additional file 7:
**Supplementary information for bioinformatic methods used for** **RNA-seq** **data analysis.** Complete descriptions and parameters of algorithms used and in-house Perl scripts for RNA-seq data processing. (DOC 87 kb)
