# Supplementary material for: Plasmodium parasites mount an arrest response to dihydroartemisinin, as revealed by whole transcriptome shotgun sequencing (RNA-seq) and microarray study
Source: BMC Genomics. 2015 Oct 21;16:830. doi: 10.1186/s12864-015-2040-0 (PMC4618149; doi:10.1186/s12864-015-2040-0)
Supplement: Additional file 3: — Morphology of treated parasites. Microscopic images were obtained from Giemsa-stained smears of P. falciparum synchronized cultures. Scale bars, 5 microns. Parasite counts of each morphologically identifiable stage (ring, troph, schizont) were determined from each experimental condition at harvest after the 1 h treatment period. (PPT 3068 kb) [file 12864_2015_2040_MOESM3_ESM.ppt]

## Slide 1
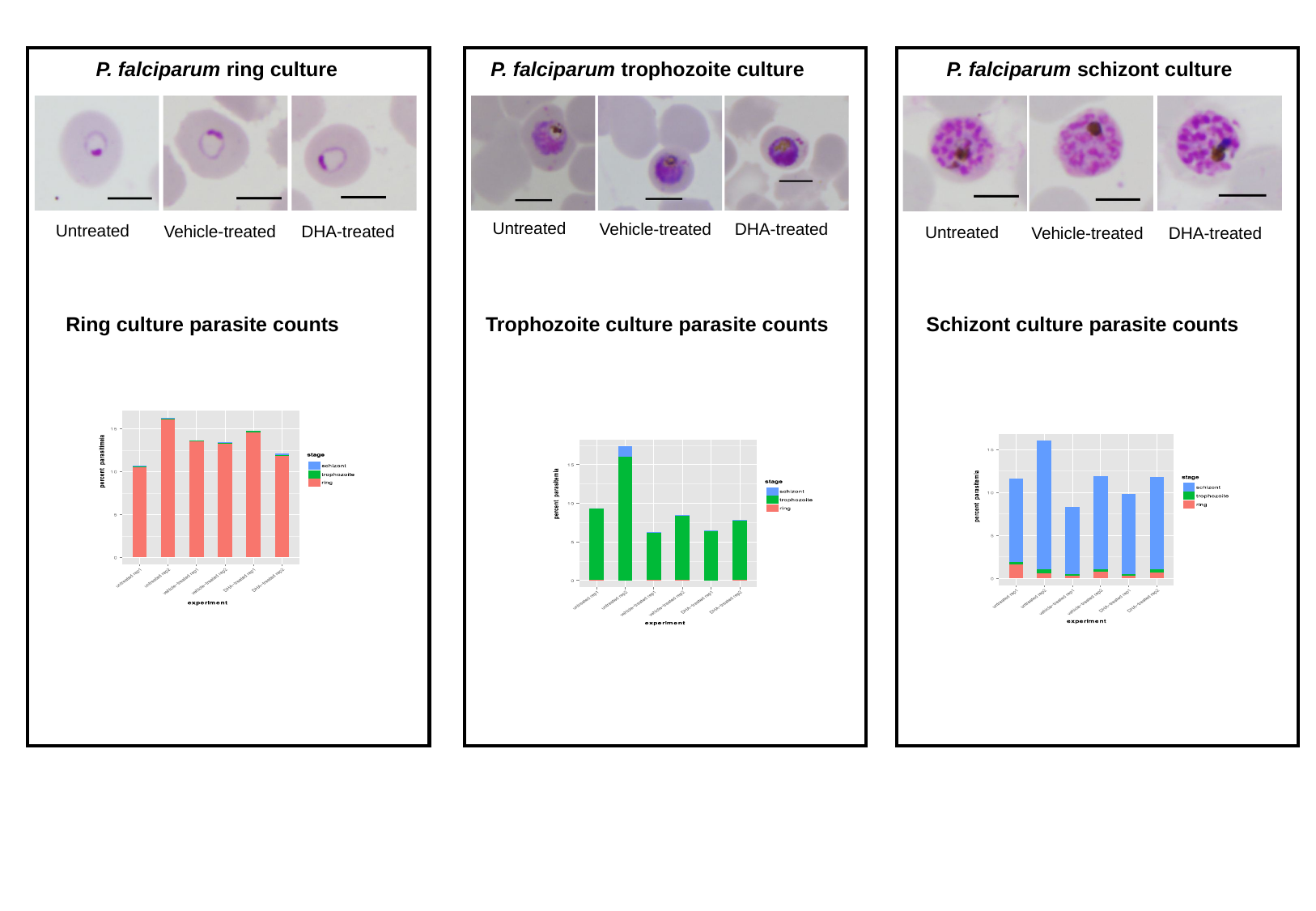

P. falciparum ring culture
P. falciparum trophozoite culture
P. falciparum schizont culture
Untreated
Vehicle-treated
DHA-treated
Untreated
Vehicle-treated
DHA-treated
Untreated
Vehicle-treated
DHA-treated
Ring culture parasite counts
Trophozoite culture parasite counts
Schizont culture parasite counts
