# Supplementary material for: Plasmodium parasites mount an arrest response to dihydroartemisinin, as revealed by whole transcriptome shotgun sequencing (RNA-seq) and microarray study
Source: BMC Genomics. 2015 Oct 21;16:830. doi: 10.1186/s12864-015-2040-0 (PMC4618149; doi:10.1186/s12864-015-2040-0)
Supplement: Additional file 7: — Supplementary information for bioinformatic methods used for RNA-seq data analysis. Complete descriptions and parameters of algorithms used and in-house Perl scripts for RNA-seq data processing. (DOC 87 kb) [file 12864_2015_2040_MOESM7_ESM.doc]

**RNA-seq data analysis methods**

## Program references:

program : prinseq-lite-0.20.3

Schmieder R and Edwards R (2011): Quality control and preprocessing of metagenomic datasets. *Bioinformatics*, **27**:863-864.

program : ncbi-blast-2.2.25+

Altschul, S.F., Gish, W., Miller, W., Myers, E.W. & Lipman, D.J. (1990) "Basic local alignment search tool." *J. Mol. Biol*. **215**:403-410

program : Tophat2

Trapnell, C., Pachter, L. & Salzberg, S.L. (2009). TopHat: discovering splice junctions with RNA-Seq. Bioinformatics **25**, 1105–1111.

Kim, D. et al. (2013) TopHat2: accurate alignment of transcriptomes in the presence of insertions, deletions and gene fusions. *Genome Biol* **14**, R36.

program : samtools

Li, Handsaker, Wysoker., Fennell., Ruan., Homer., Marth., Abecasis., Durbin R. and 1000 Genome Project Data Processing Subgroup (2009). The Sequence alignment/map (SAM) format and SAMtools. *Bioinformatics* **25**, 2078-9.

**Pre-processing**

1. **Remove rRNA reads**

- convert input fastq to fasta

program : prinseq-lite-0.20.3

parameters:

-out_format 1

- obtain rRNA sequences (fasta) from NCBI
- blastn input with rRNA sequences

program : ncbi-blast-2.2.25+

parameters:

-evalue 1e-4

-perc_identity 90

-outfmt 6

-num_threads 8

- identify sequences that map with rRNA, then remove from input

**2. Trim low-quality base-calls and remove bad reads**

- trim N

program : prinseq-lite-0.20.3

parameter :

-trim_ns_left 2 : trim polyN from 5′ if N more than 2

-trim_ns_right 2 : trim polyN from 3′ if N more than 2

- filter NN in middle of read

Count base until NN is found, then stop and keep distal bases.

- filter quality

program: prinseq-lite-0.20.3

parameters:

-min_qual_mean 20 : filter mean quality of read minimum 20

-derep 1 : remove exact duplicates

**Mapping process**

Map reads to reference genome using Tophat2 with Bowtie2 programs.

parameters:

--max-insertion-length 0 : not allow insertion

--max-deletion-length 0 : not allow deletion

--read-mismatches 10 : allow mismatch 10 positions

--read-gap-length 0 : not allow gap

--read-edit-dist 10 : allow edit base 10 positions

-i 10 : minimum intron length (vary by species)

-l 2425 : maximum intron length (vary by species)

get length intron by GFF annotation of *Plasmodium* version 11

*Plasmodium berghei* max = 2432 min = 1

*Plasmodium falciparum* 3D7 max = 1360 min = 1

-g 1 : unique mapping

-p 8 : process to run

**Post processing**

Remove potential duplicates and inaccurately mapped reads.

1. Remove potential PCR duplicates

program : samtools

parameters:

sort : sort bam file

rmdup -s : remove duplicates for paired end reads

2. Remove inaccurately mapped reads

Adjust number of allowed mismatches according to trimmed read length:

35-50 bases allow max 1 mismatch,

51-100 bases allow max 2 mismatches,

101+ bases allow max 3 mismatches

programs: samtools, custom perl script separateRead2.pl

parameters:

calmd :calculate MD tag for present no. mismatch

3. Filter quality of reads

program :perl script findquality_samfile.pl

1. extract properly paired, read1, read2, forward and reverse mapped reads

program: samtools

parameters:

view -h -f 0x0040 : read1

view -h -f 0x0080 : read2

view -F 0x10 : forward

view -f 0x10 : reverse

**Calculate read counts for each gene and RPKM**

Use samtools to extract reads in the range of all annotated exons of each gene, separate reads between sense and antisense then calculate RPKM for each gene

program: samtools, custom perl script

**Perl script for calculating sense/antisense counts**

input: forward read bamfile, reverse read bamfile

process:

get detail of annotated genes from PlasmoDB database -> count forward read and reverse read that rely on range of each exon by using samtools “samtools view bamfile coordinate(chr:x-y) | wc -l” , then merge amount of forward read and reverse read of every exon

next, if gene is located on the plus strand, sense read count is the total of mapped forward reads and antisense is the total of reverse reads, and vice versa for genes orientated on the minus strand.

#!/usr/bin/perl -w

use strict;

use DBI;

use DBD::mysql;

#use Mysql;

my $bamfileforward = $ARGV[0]; #bamfile of forward mapping read

my $bamfilereverse = $ARGV[1]; #bamfile of reverse mapping read

## connect mySQL database for collect annotation data

my $database = "plasmodium falciparum database from GFF of plasmodium falciparum version 11 (www.plasmoDB.org)";

my $dsn = "dsn of mySQL";

my $user = "user";

my $pw = "password";

my $connect = DBI->connect($dsn,$user,$pw)

or die "Connection Error: $DBI::errstr\n";

# select sample_name from gene_on_Genome GROUP BY `sample_name` #each sample_name

my $query_sample_name = "select * from gff_genes_info_11_Pfalciparum3D7";

my $query_handle_sp = $connect->prepare($query_sample_name);

my %gene = ();

my %result = ();

$query_handle_sp->execute or die "SQL Error: $DBI::errstr\n";

while(my $hash_ref = $query_handle_sp->fetchrow_hashref)

{

my $id = $hash_ref->{id};

$gene{$id}{"gene_name"} = $hash_ref->{gene_name};

$gene{$id}{"description"} = $hash_ref->{description};

$gene{$id}{"Ref_seq"} = $hash_ref->{seq_id};

my $chro = $gene{$id}{"Ref_seq"};

$gene{$id}{"start"} = $hash_ref->{phys_start};

$gene{$id}{"end"} = $hash_ref->{phys_end};

$gene{$id}{"strand"} = $hash_ref->{strand};

$gene{$id}{"exon_count"} = $hash_ref->{exon_count};

$gene{$id}{"exon_starts"} = $hash_ref->{exon_starts};

$gene{$id}{"exon_ends"} = $hash_ref->{exon_ends};

## collect data in each exon

my @tmpstart = split(/,/, $gene{$id}{"exon_starts"});

my @tmpend = split(/,/, $gene{$id}{"exon_ends"});

my $length = 0;

my $lengthall = 0;

my $lengthlong = 0;

my $countreadlongforward = 0;

my $countreadlongreverse = 0;

my $coordinate;

my $countreadforward = 0;

my $countallforward = 0;

my $countreadreverse = 0;

my $countallreverse = 0;

################ count read only exon range ##################################

for(my $i = 0; $i < $gene{$id}{"exon_count"};$i++)

{

$length = ($tmpend[$i]-$tmpstart[$i])+1;

$coordinate = "$chro:$tmpstart[$i]-$tmpend[$i]";

$countreadforward = qx(samtools view $bamfileforward $coordinate | wc -l);

chomp($countreadforward);

$lengthall = $lengthall+$length;

$countallforward = $countallforward+$countreadforward;

$countreadreverse = qx(samtools view $bamfilereverse $coordinate | wc -l);

chomp($countreadreverse);

$countallreverse = $countallreverse+$countreadreverse;

}

my $C_value_short_fwd = $countallforward;

my $C_value_short_rev = $countallreverse;

my $L_value_short = $lengthall/1000;

########### if gene have more structure then check duplicate and choose longest range ######################

if (exists $result{$gene{$id}{"gene_name"}})

{

if($result{$gene{$id}{"gene_name"}}{"lengthshort"}<$L_value_short)

{

$result{$gene{$id}{"gene_name"}}{"lengthshort"} = $L_value_short;

$result{$gene{$id}{"gene_name"}}{"countshortfwd"} = $C_value_short_fwd;

$result{$gene{$id}{"gene_name"}}{"countshortrev"} = $C_value_short_rev;

$result{$gene{$id}{"gene_name"}}{"exonstart"} = $gene{$id}{"exon_starts"};

$result{$gene{$id}{"gene_name"}}{"exonend"} = $gene{$id}{"exon_ends"};

$result{$gene{$id}{"gene_name"}}{"chr"} = $gene{$id}{"Ref_seq"};

$result{$gene{$id}{"gene_name"}}{"strand"} = $gene{$id}{"strand"};

}

elsif($result{$gene{$id}{"gene_name"}}{"lengthshort"}==$L_value_short)

{

if($result{$gene{$id}{"gene_name"}}{"countshortfwd"}< $C_value_short_fwd && $result{$gene{$id}{"gene_name"}}{"countshortrev"}< $C_value_short_rev)

{

$result{$gene{$id}{"gene_name"}}{"lengthshort"} = $L_value_short;

$result{$gene{$id}{"gene_name"}}{"countshortfwd"} = $C_value_short_fwd;

$result{$gene{$id}{"gene_name"}}{"countshortrev"} = $C_value_short_rev;

$result{$gene{$id}{"gene_name"}}{"exonstart"} = $gene{$id}{"exon_starts"};

$result{$gene{$id}{"gene_name"}}{"exonend"} = $gene{$id}{"exon_ends"};

$result{$gene{$id}{"gene_name"}}{"chr"} = $gene{$id}{"Ref_seq"};

$result{$gene{$id}{"gene_name"}}{"strand"} = $gene{$id}{"strand"};

}

}

}

else

{

$result{$gene{$id}{"gene_name"}}{"lengthshort"} = $L_value_short;

$result{$gene{$id}{"gene_name"}}{"countshortfwd"} = $C_value_short_fwd;

$result{$gene{$id}{"gene_name"}}{"countshortrev"} = $C_value_short_rev;

$result{$gene{$id}{"gene_name"}}{"exonstart"} = $gene{$id}{"exon_starts"};

$result{$gene{$id}{"gene_name"}}{"exonend"} = $gene{$id}{"exon_ends"};

$result{$gene{$id}{"gene_name"}}{"chr"} = $gene{$id}{"Ref_seq"};

$result{$gene{$id}{"gene_name"}}{"strand"} = $gene{$id}{"strand"};

}

}

## print to file

my $ratiosense_antisense = 0;

for my $key ( sort keys %result )

{

if($result{$key}{'strand'} eq "+")

{

$ratiosense_antisense = $result{$key}{'countshortfwd'}/$result{$key}{'countshortrev'};

print "$key\t$result{$key}{'chr'}\t$result{$key}{'strand'}\t$result{$key}{'lengthshort'}\t$result{$key}{'countshortfwd'}\t$result{$key}{'countshortrev'}\t$ratiosense_antisense\n";

}

elsif($result{$key}{'strand'} eq "-")

{

$ratiosense_antisense = $result{$key}{'countshortrev'}/$result{$key}{'countshortfwd'};

print "$key\t$result{$key}{'chr'}\t$result{$key}{'strand'}\t$result{$key}{'lengthshort'}\t$result{$key}{'countshortrev'}\t$result{$key}{'countshortfwd'}\t$ratiosense_antisense\n";

}

}

**perl script for calculating RPKM**

input: bamfile, amount of all mapped reads

process: get details of annotated genes from PlasmoDB database

for each gene -> count read that rely on range of each exon by using samtools “samtools view bamfile coordinate(chr:x-y) | wc -l” , then merge read counts of all exons.

Next, calculate RPKM = total mapped reads to gene / (total of all mapped reads/1000000) * (length of all exons combined/1000)

#!/usr/bin/perl -w

use strict;

use DBI;

use DBD::mysql;

#use Mysql;

my $bamfile = $ARGV[0]; #bamfile of all mapping read

my $allmapread = $ARGV[1]; #amount of all read

my $N_value = $allmapread/1000000;

## connect mySQL database for collect annotation data

my $database = "plasmodium falciparum database from GFF of plasmodium falciparum version 11 (www.plasmoDB.org)";

my $dsn = "dsn of mySQL";

my $user = "user";

my $pw = "password";

my $connect = DBI->connect($dsn,$user,$pw)

or die "Connection Error: $DBI::errstr\n";

my $query_sample_name = "select * from gff_genes_info_11_Pfalciparum3D7";

my $query_handle_sp = $connect->prepare($query_sample_name);

my %gene = ();

my %result = ();

$query_handle_sp->execute or die "SQL Error: $DBI::errstr\n";

while(my $hash_ref = $query_handle_sp->fetchrow_hashref)

{

my $id = $hash_ref->{id};

$gene{$id}{"gene_name"} = $hash_ref->{gene_name};

$gene{$id}{"Ref_seq"} = $hash_ref->{seq_id};

my $chro = $gene{$id}{"Ref_seq"};

$gene{$id}{"start"} = $hash_ref->{phys_start};

$gene{$id}{"end"} = $hash_ref->{phys_end};

$gene{$id}{"strand"} = $hash_ref->{strand};

$gene{$id}{"exon_count"} = $hash_ref->{exon_count};

$gene{$id}{"exon_starts"} = $hash_ref->{exon_starts};

$gene{$id}{"exon_ends"} = $hash_ref->{exon_ends};

## collect data in each exon

my @tmpstart = split(/,/, $gene{$id}{"exon_starts"});

my @tmpend = split(/,/, $gene{$id}{"exon_ends"});

my $length = 0;

my $lengthall = 0;

my $lengthlong = 0;

my $countreadlong = 0;

my $coordinate;

my $countread = 0;

my $countall = 0;

############ count read in all range (include exon and intron) ###########

my $last = $gene{$id}{"exon_count"}-1;

$lengthlong = ($tmpend[$last]-$tmpstart[0])+1;

my $L_value_long = $lengthlong/1000;

$coordinate = "$chro:$tmpstart[0]-$tmpend[$last]";

$countreadlong = qx(samtools view $bamfile $coordinate | wc -l);

chomp($countreadlong);

my $C_value_long = $countreadlong;

my $RPKM_long = $C_value_long/($N_value*$L_value_long);

################ count read only exon range ##################################

for(my $i = 0; $i < $gene{$id}{"exon_count"};$i++)

{

$length = ($tmpend[$i]-$tmpstart[$i])+1;

$coordinate = "$chro:$tmpstart[$i]-$tmpend[$i]";

$countread = qx(samtools view $bamfile $coordinate | wc -l);

chomp($countread);

$lengthall = $lengthall+$length;

$countall = $countall+$countread;

}

my $C_value_short = $countall;

my $L_value_short = $lengthall/1000;

my $RPKM_short = $C_value_short/($N_value*$L_value_short);

########### if gene have more structure then check duplicate and choose longest range ######################

if (exists $result{$gene{$id}{"gene_name"}})

{

if($result{$gene{$id}{"gene_name"}}{"lengthshort"}<$L_value_short)

{

$result{$gene{$id}{"gene_name"}}{"lengthshort"} = $L_value_short;

$result{$gene{$id}{"gene_name"}}{"lengthlong"} = $L_value_long;

$result{$gene{$id}{"gene_name"}}{"countshort"} = $C_value_short;

$result{$gene{$id}{"gene_name"}}{"countlong"} = $C_value_long;

$result{$gene{$id}{"gene_name"}}{"RPKMshort"} = $RPKM_short;

$result{$gene{$id}{"gene_name"}}{"RPKMlong"} = $RPKM_long;

$result{$gene{$id}{"gene_name"}}{"exonstart"} = $gene{$id}{"exon_starts"};

$result{$gene{$id}{"gene_name"}}{"exonend"} = $gene{$id}{"exon_ends"};

$result{$gene{$id}{"gene_name"}}{"chr"} = $gene{$id}{"Ref_seq"};

}

elsif($result{$gene{$id}{"gene_name"}}{"lengthshort"}==$L_value_short)

{

if($result{$gene{$id}{"gene_name"}}{"countshort"}< $C_value_short)

{

$result{$gene{$id}{"gene_name"}}{"lengthshort"} = $L_value_short;

$result{$gene{$id}{"gene_name"}}{"lengthlong"} = $L_value_long;

$result{$gene{$id}{"gene_name"}}{"countshort"} = $C_value_short;

$result{$gene{$id}{"gene_name"}}{"countlong"} = $C_value_long;

$result{$gene{$id}{"gene_name"}}{"RPKMshort"} = $RPKM_short;

$result{$gene{$id}{"gene_name"}}{"RPKMlong"} = $RPKM_long;

$result{$gene{$id}{"gene_name"}}{"exonstart"} = $gene{$id}{"exon_starts"};

$result{$gene{$id}{"gene_name"}}{"exonend"} = $gene{$id}{"exon_ends"};

$result{$gene{$id}{"gene_name"}}{"chr"} = $gene{$id}{"Ref_seq"};

}

}

}

else

{

$result{$gene{$id}{"gene_name"}}{"lengthshort"} = $L_value_short;

$result{$gene{$id}{"gene_name"}}{"lengthlong"} = $L_value_long;

$result{$gene{$id}{"gene_name"}}{"countshort"} = $C_value_short;

$result{$gene{$id}{"gene_name"}}{"countlong"} = $C_value_long;

$result{$gene{$id}{"gene_name"}}{"RPKMshort"} = $RPKM_short;

$result{$gene{$id}{"gene_name"}}{"RPKMlong"} = $RPKM_long;

$result{$gene{$id}{"gene_name"}}{"exonstart"} = $gene{$id}{"exon_starts"};

$result{$gene{$id}{"gene_name"}}{"exonend"} = $gene{$id}{"exon_ends"};

$result{$gene{$id}{"gene_name"}}{"chr"} = $gene{$id}{"Ref_seq"};

}

}

## print to file

for my $key ( sort keys %result )

{

print "$key\t$result{$key}{'chr'}\t$result{$key}{'lengthshort'}\t$result{$key}{'countshort'}\t$result{$key}{'RPKMshort'}\t$result{$key}{'lengthlong'}\t$result{$key}{'countlong'}\t$result{$key}{'RPKMlong'}\t$result{$key}{'exonstart'}\t$result{$key}{'exonend'}\n";

}
